# Key Conditions for Successful Implementation of the Manchester Procedure as Primary Surgical Treatment for Mild to Moderate Uterine Prolapse: A Qualitative Study Among Dutch Gynecologists

**DOI:** 10.1007/s00192-026-06525-7

**Published:** 2026-02-07

**Authors:** Lisa M. Stoter, Eva W. Verkerk, Kim J. B. Notten, Simone A. van Dulmen, Kirsten B. Kluivers

**Affiliations:** 1 Department of Obstetrics and Gynecology, Radboud University Medical Center, Geert Grooteplein Zuid 10, 6525 GA Nijmegen, The Netherlands; 2 Science Department IQ Health, Radboud University Medical Center, Nijmegen, The Netherlands

**Keywords:** Implementation, Manchester procedure, Sacrospinous hysteropexy, Pelvic organ prolapse

## Abstract

**Introduction and Hypothesis:**

Recent evidence supports the clinical and economic advantages of the Manchester procedure (MP) over sacrospinous hysteropexy (SSH) in women treated for mild to moderate uterine prolapse. However, its use remains inconsistent, resulting in a notable variation in current practice. This study explores gynecologists’ barriers and facilitators to adoption of the MP, aiming to guide the development of a targeted implementation strategy.

**Methods:**

From March to June 2025, we conducted semi-structured interviews with gynecologists (*n* = 12) from the Netherlands in different stages of implementation to identify barriers and facilitators. The updated Consolidated Framework for Implementation Research (CFIR) guided data collection and analysis, ensuring a structured assessment of key domains.

**Results:**

A total of 12 barriers and 20 facilitators were identified in 13 constructs across all CFIR domains. Key facilitators for implementation included strong regional collaboration networks, the low complexity of the surgical technique, peer-driven motivation to align with national trends, and overall trust in the evidence, although the latter varied between clinicians. Main barriers were the personal and organizational effort required for training and implementation, particularly in the absence of an experienced colleague, satisfaction with SSH results, and concerns about lower reimbursement for the MP.

**Conclusions:**

This study identified key barriers and facilitators influencing the adoption of the MP from the perspective of gynecologists, emphasizing the clinical, organizational, and financial factors involved. Addressing these barriers and leveraging facilitators could enhance adoption of the MP, potentially improving treatment outcomes and reducing healthcare costs.

**Supplementary Information:**

The online version contains supplementary material available at 10.1007/s00192-026-06525-7.

## Introduction

Pelvic organ prolapse (POP) is a prevalent condition adversely affecting women’s quality of life [1]. Given the high prevalence and the potential impact on healthcare resources, it is crucial that treatment options for POP are both clinically effective and economically sustainable. Of the two uterus-preserving surgical treatments, the Manchester procedure (MP) has been reported to achieve better surgical outcomes and appears more cost-effective than sacrospinous hysteropexy (SSH) in patients with mild to moderate uterine prolapse, based on available evidence from the Netherlands and the UK [2–4].

Despite growing evidence for best practices, variation in surgical treatment for uterine prolapse remains substantial [5, 6]. The uptake of new evidence into clinical practice is often slow, and clinical decisions may vary widely between clinicians [7, 8]. Prior to the availability of robust evidence, gynecologists based their decision between the MP and SSH on individual experience and training [9]. This contributed to the practice pattern variation across Dutch hospitals, potentially leading to unequal access to effective care and unnecessary costs [5]. The randomized clinical trial comparing the MP and SSH, the SAM study [3], was set up to promote more standardized care, but it does not appear to have reduced this variation. To date, it remains unclear why the MP is more frequently used in some hospitals than in others. Implementation research in urogynecology is scarce, while identifying the conditions under which implementation succeeds is essential for the development of effective, context-specific strategies [10]. Gaining insight into gynecologists’ perspectives on the MP following the publication of the SAM study is particularly important, given their central role in clinical decision-making and their influence on national protocols and policies. These insights are crucial for optimizing care within the Dutch healthcare system. This study also offers valuable perspectives for international settings facing similar challenges.

The aim of this interview study is to identify factors that facilitate or hinder the implementation of the MP from the perspective of gynecologists. We seek to uncover the conditions under which adoption of the MP succeeds and to identify which barriers still need to be addressed.

## Methods

### Study Design

This is a qualitative study exploring barriers and facilitators to the implementation of the MP, based on semi-structured interviews with Dutch gynecologists. The study was reported according to the Consolidated criteria for Reporting Qualitative research (COREQ) checklist (Appendix I) [11].

### Study Population

Eligible participants were Dutch gynecologists performing vaginal surgery, working in hospitals that had implemented the MP, were in the process, or had not yet adopted it. Participants were recruited through purposive sampling to ensure representation from hospitals at different stages of implementation, based on national benchmarking data from 2024 [12]. During recruitment, we discovered that some gynecologists had since adopted the MP despite being classified as non-users in the benchmark data. To address this and avoid bias toward experienced users, we adapted our recruitment strategy by specifically targeting gynecologists with limited or no experience with MP through our and other participants’ professional network. With this approach we allowed for a comprehensive exploration of perspectives across implementation contexts. Recruitment was conducted in collaboration with the pelvic floor disorders subcommittee of the Dutch Society for Obstetrics and Gynecology. We also aimed for diversity in individual characteristics, hospital types (academic, teaching, and general), and geographic regions. Eligible participants received an email with study information and an invitation to participate. During interviews, participants were asked to identify gynecologists with or without experience with the MP to support further recruitment. Of nineteen clinicians approached, 12 participated (response rate 63%). Five clinicians did not respond to email invitations, and two were excluded because they performed many MPs and this group was already well represented. After the tenth interview, data saturation was reached. At this point, we found that themes were fully developed and a diverse range of views, both supportive and critical, were captured [13]. No new themes emerged during the final two interviews, indicating that further data collection was unlikely to provide additional insights. Prior to the interview, participants could ask questions after reading the informed consent form. They were assured that the data collected would not be traceable to individual persons. All gave orally recorded consent.

### Data Collection

All interviews were conducted by one or two researchers (LS and EV) and took place in a video call via Microsoft Teams. Interviews took place from March to June 2025. The interview guide was developed on the basis of the updated Consolidated Framework for Implementation Research (CFIR) and the CFIR Interview Guide Tool to ensure a comprehensive exploration of factors influencing implementation [14]. This methodological foundation allowed for a systematic evaluation of barriers and facilitators relevant to the study’s context. Questions were translated into Dutch and adapted to the surgical context. To minimize bias, we used open-ended, neutrally phrased questions and encouraged participants to elaborate freely. The full interview guide can be found in Appendix II. Data on baseline characteristics of participants was collected during the interview.

### Data Analysis

All interviews were digitally recorded, anonymized and transcribed verbatim. Data were analyzed using ATLAS.ti (version 24.0.0) following a systematic approach starting with open coding. Initially, two researchers (LS and EV) both independently coded the first five interview transcripts after which codes were compared and refined through consensus. Since by the fifth interview, the preliminary coding framework was established and our coding was reasonably consistent, the remaining interviews were divided between the researchers for initial coding, with the final three coded by LS and a third researcher (SD). Throughout the process, codes were regularly discussed and adjusted to ensure consistency. Two coders (EV and SD) had extensive experience with qualitative research and semi-structured interviews, while the third (LS) had basic experience. To ensure reliability, all coding by the less experienced coder was reviewed by an experienced team member. After open coding, codes were grouped into broader themes using the CFIR [14]. This framework served as a guiding structure for categorization and ensured alignment with key constructs. The final coding structure was refined through ongoing team discussions and validation.

### Ethical Approval

The medical ethics committee concluded that this study did not require ethical approval under Dutch national law.

## Results

Saturation of data was achieved after 12 interviews. An overview of the baseline characteristics of participants is provided in Table 1. Interviews had a median duration of 34 min, ranging from 27 to 68 min. Four participants already performed the MP regularly before or during the research phase of the SAM study, four started implementation as a result of the SAM study, and four did not routinely offer the MP as standard treatment for patients with mild to moderate prolapse or did not perform it at all.

**Table 1:** Baseline demographics of gynecologists participating in the interviews

	Participants *n* = 12
Age in years (median, range)	42 (35–66)
Sex (*n*, %)	
Female	9 (75)
Practice type (*n*, %)	
Academic teaching hospital Non-academic teaching hospital Non-teaching hospital	1 (8) 2 (17) 9 (75)
Years since completing residency (median, range)	9 (1–29)
Region of residency (*n*, %)	
Northern Netherlands Central Netherlands Southern Netherlands	2 (17) 6 (50) 4 (33)
Learned the MP during residency	
Yes (*n*, %)	7 (58)
Formal urogynecology accreditation^a^ (*n*, %)	
Yes	3 (25)
Experience with the MP per gynecologist per year (reference year 2024) (*n*, %)	
0 1–25 >25	3 (25) 6 (50) 3 (25)
Most frequently performed uterine prolapse procedure (reference year 2024) (*n*, %)	
MP SSH VH	6 (50%) 5 (42%) 1 (8%)

*n* number (of participants), *MP* Manchester procedure, *SSH* sacrospinous hysteropexy, *VH* vaginal hysterectomy

^a^Subspecialty accreditation in urogynecology has been officially recognized by the Dutch association of Obstetrics & Gynecology since 2009, but it is not mandatory for practicing gynecologists [15]

A total of 12 barriers and 20 facilitators were identified in 13 constructs across all CFIR domains. Table 2 provides an overview of the identified constructs and their alignment with the respective CFIR domains. To facilitate a deeper understanding of the implementation dynamics, the identified constructs are discussed below.

**Table 2:** Barriers and facilitators for implementation of the Manchester procedure as primary surgical treatment for women with mild to moderate uterine prolapse, organized by CFIR domains and constructs

CFIR constructs		
*Characteristics of the procedure*	*Barriers*	*Facilitators*
Complexity of the procedure		• Technique resembles vaginal hysterectomy
Quality of the evidence	• Perceived insufficiency of evidence supporting the MP	• Trust in evidence supporting the MP
Relative advantage	• Satisfaction with current practice	• Perceived clinical benefits of the MP
Applicability in patients	• The MP is not suitable for severe and vault prolapse, whereas SSH is applicable across more indications • Concerns about diagnostic limitations after the MP	• Anatomical cutoff values from the SAM study provide guidance for selecting between the MP and SSH
*Inner setting*	*Barriers*	*Facilitators*
Regional collaboration networks		• Regional collaboration networks offering peer learning and hands-on training • Information sharing through regional network meetings • Access to experienced colleagues within or outside the hospital
Local expertise and training infrastructure	• Need for personal initiative to arrange external training • Training outside working hours seen as time-consuming • Lower reimbursement for the MP may impact hospital finances	• Training during working hours • Patient care prioritized over financial concerns
Financial consequences		• Similar logistics and materials for the MP and SSH
Available recourses		• Supportive team culture and peer influence
Influence of colleagues		• Presence of a local champion to lead implementation
*Outer setting*	*Barriers*	*Facilitators*
Institutional role of the professional association		• Subcommittee^a^ provides visibility for new evidence • Potential role for subcommittee^a^ in connecting trainers and trainees
Role of the clinical guideline	• Lack of guideline recommendation for the MP in guideline generally seen as a limited barrier	
*Characteristics of individuals*	*Barriers*	*Facilitators*
Competency and learning	• Anticipated relevant time investment for learning the MP • Need for sufficient patient volume post-training to retain skills • Low case numbers hinder skill proficiency • Cautiousness toward rapid adoption of new evidence	• Perceived short learning curve • Learned the MP technique during residency
Attitude toward new surgical techniques		• Interest in new techniques • Sense of duty to offer best patient care
Sense of obligation		• Peer-driven desire to stay aligned with national trends
*Implementation process*	*Barriers*	*Facilitators*
Timing		• Perceived national momentum

*CFIR* updated Consolidated Framework for Implementation Research, *MP* Manchester procedure, *SSH* sacrospinous hysteropexy

^a^Subcommittee on pelvic floor disorders of the Dutch Society for Obstetrics and Gynecology

### Characteristics of the Procedure

#### Complexity of the Procedure

Most gynecologists considered the MP a relatively easy technique to learn and to supervise, due to its similarity to vaginal hysterectomy. Among those who had not yet mastered the procedure, one gynecologist expressed concern that the procedure might be difficult to learn. Complications like intra-operative hemorrhage were mentioned, but gynecologists generally felt confident in managing these themselves. Overall, this perceived ease was seen as a facilitator for its adoption.

It is sometimes said that if you can perform a vaginal hysterectomy, you can almost perform an MP as well. Not entirely, but you’re pretty close. – participant 2

#### Quality of the Evidence

The majority of the participants expressed confidence in the quality and outcomes of the SAM study, which inspired some directly to adopt the MP. A few raised concerns about basing clinical decisions solely on the SAM study, especially given the difference in surgical success rates after SSH between the SAM and SAVE U study [3, 16]. In the SAVE U, the SSH performed equal to the MP in the SAM study. Some interviewees perceived their own SSH outcomes as superior to those reported in the SAM study, which influenced their interpretation of the evidence. Consistent international findings and long-term follow-up data were identified as potential facilitators for future implementation. The cost differences between the MP and SSH had not yet been published during the interviews.

In my experience, there is little to no debate regarding the outcomes of the SAM study.” – participant 3

I belong to the group of gynecologists who achieve results similar [with SSH] to those reported in the SAVE U trial. (…) So, based on the findings of the SAM trial, I don’t see any reason to change my current clinical approach. – participant 7

#### Relative Advantage

Participants generally viewed the MP as advantageous over SSH in cases of mild to moderate uterine prolapse, citing better surgical outcomes, a lower anterior compartment recurrence and absence of post-operative buttock pain as key advantages. Disadvantages of the MP included difficulties in diagnosing postmenopausal bleeding and endometrial cancer due to cervical stenosis, although these concerns were only raised by interviewees not yet performing the procedure. Some participants felt little urgency to adopt the MP, as they considered SSH faster and sufficiently effective in their hands.

If you’re satisfied with the technique you have (SSH), it makes it harder to introduce something new in its place. – participant 6

#### Applicability in Patients

A commonly cited barrier was the perceived limited applicability of the MP in cases of more severe stages of uterine descent and vaginal vault prolapse, whereas SSH has a wider indication. In contrast, participants who adopted the MP identified the anatomical cutoff values from the SAM study as a facilitator, providing clear guidance for selecting between the MP and SSH and supporting its use in the majority of patients.

It must be technically feasible, right? I fully agree that when there is a total prolapse, the MP is not the appropriate choice, and that SSH is likely a much better option. – participant 7

### Inner Setting

#### Regional Collaboration Networks

Within the inner setting domain, regional collaboration networks were widely seen as key facilitators, offering learning opportunities through meetings, peer connections, and hands-on MP training. A majority of participants first heard about the SAM study through these meetings, which they described as a stimulus for adoption. However, not all gynecologists were involved in such networks, and they tended to perceive limited added value in regional collaboration. Some participants felt existing national education was already sufficient.

So I already knew them [regional colleagues] well, and they had mentioned before that if I was interested, I could come and perform the MP with them. So, yeah, the threshold to do it was really low. I felt really welcome. – participant 3

#### Local Expertise and Training Infrastructure

A key barrier for gynecologists not yet performing the MP was the absence of a colleague within their hospital to provide hands-on training, requiring personal initiative to seek external mentorship. Concerns were also raised about lacking senior backup during procedures and the confidence needed to manage complications independently. In contrast, having an experienced colleague within one’s network was seen as a strong facilitator. Positively, all interviewees reported having access to someone in their network from whom they could learn the procedure. Training during working hours was available to approximately half of the participants, others had to arrange this in their free time. Although the time investment necessary for this discouraged some, it was generally seen as a minor barrier and several participants nevertheless pursued training on their own initiative.

They [regional colleagues] scheduled four MP’s in one day, so that also requires something from them in terms of planning. (…) So yes, that naturally takes some effort, because I also need to have a day when I can go there and things like that. – participant 11

#### Financial Consequences

Approximately half of participants were aware that reimbursement by health care insurers for the MP is lower than for SSH, despite similar costs associated with the surgical procedure and hospital admission. While they agreed that this discrepancy should be addressed to ensure fair compensation, only a minority considered it a true barrier to implementation. Most gynecologists emphasized that financial factors should not outweigh the priority of delivering high-quality patient care.

Well, that’s a barrier, yes. (…) Financially, it’s not attractive for the hospital to perform an MP. Yes, I do see that as a problem. - participant 4

#### Available Recourses

A facilitating factor was the similarity in logistics and materials between the MP and SSH, with recent adopters reporting minimal logistic issues. Participants often arranged material lists and surgical reports through their own professional networks. Most indicated that having such resources publicly available would facilitate implementation. A minor barrier mentioned was a temporary decrease in efficiency when starting with the MP due to more time being scheduled in, although this was expected to improve quickly with experience.

For the MP, you don’t really need any other materials, right? You just use the basic set, the same one you would use for anterior or posterior colporrhaphy. So, no, logistics was not a problem. – participant 8.

#### Influence of Colleagues

A supportive team environment was an important facilitator for implementation of the MP. Most gynecologists reported regular team discussions of new evidence and alignment of practices. A key facilitator was the involvement of a local champion to initiate and lead adoption; in its absence, progress was limited despite openness for change among colleagues. The surgical preferences of direct colleagues were reported to influence individual choices, as gynecologists often train each other and adopted techniques commonly used within their team. Some teams have no established agreements on which surgical procedure should be used for which type of patient, sometimes leading to variability in practice within a hospital.

### Outer Setting

#### Institutional Role of the Professional Association

In the outer setting domain, the existence of the pelvic floor disorders subcommittee of the Dutch Society for Obstetrics and Gynecology was perceived as a facilitating factor for implementation. Some gynecologists recognized potential roles for this subcommittee in the implementation process, such as connecting clinical trainers with hospitals, serving as a platform for discussion and expert opinion, and providing visibility for new evidence through symposia. The subcommittee has already discussed the SAM study on several occasions, which many gynecologists cited as their main source of familiarity with the results.

I believe the subcommittee could play a facilitating role in helping identify suitable trainers, particularly for those without an established professional network, by connecting individuals and supporting knowledge exchange. –participant 5

#### Role of the Clinical Guideline

The absence of a formal guideline on uterine-sparing prolapse surgeries like the MP and SSH was considered important, though not universally perceived as a barrier, as many had already initiated adoption without it. However, many participants emphasized that formal inclusion of the MP in national guidelines is a prerequisite for the pelvic floor disorders subcommittee of the Dutch Society for Obstetrics and Gynecology to actively promote its implementation.

If the guideline states that, for a certain type of prolapse, the preferred treatment is the MP, then of course you’re expected to follow that. You’d really need to have a strong case to justify deviating from it. – participant 3

### Characteristics of Individuals

#### Competency and Learning

Several gynecologists experienced the relatively short learning curve of the MP, typically one to two days of supervised practice. Prior exposure to the MP during residency was seen as a facilitating factor for adoption. In contrast, barriers included uncertainty regarding the time required to learn the procedure among those without prior experience. One gynecologist anticipated a substantial time investment, and another emphasized the need for a sufficient number of patients immediately after training to retain skills, which are organizational challenges. Additionally, opinions varied on whether current case volumes for both the MP and SSH remain sufficient to maintain surgical proficiency to deal with complications, with low volumes per procedure in smaller hospitals explicitly mentioned as a barrier by one participant.

You know, you just have to learn a new technique again and become proficient in it, which simply takes some time, and you just have to get through that. – participant 12

#### Attitude Toward New Surgical Techniques

Most gynecologists described a positive attitude toward innovation in general, viewing the opportunity to learn new surgical techniques as a facilitator. However, a few participants demonstrated a more cautious or wait-and-see approach, which acted as a barrier. They emphasized that immediate adoption of emerging evidence may be inefficient or overly driven by enthusiasm. One gynecologist referred to the rapid, later-reversed implementation of vaginal mesh procedures as a reason to remain cautious, emphasizing a preference for clinical stability over constantly shifting with new evidence.

Well, there was, and fortunately there still is, a healthy interest in new techniques, right? Sometimes it works against us when suddenly everyone started using meshes. But overall, yes, urogynecologists are still quite curious. – participant 7

#### Sense of Obligation

A key facilitator for implementation was the strong sense of professional responsibility toward patients. Several gynecologists who had already adopted the MP emphasized that not offering this procedure would mean withholding what they considered the best available care from their patients. Similarly, half of the gynecologists who had not yet adopted the MP identified this sense of duty as a potential driver for future implementation. Moreover, the majority expressed a desire to stay aligned with national trends in treatment options for POP.

And just continuing to do SSH’s because I couldn’t do the MP; I felt that, with these results, I was obligated to offer it to the patients as well. – participant 3

#### Implementation Process

A majority of the participants felt that the adoption of the MP was progressing well in the Netherlands, observing that many hospitals seem to have adopted the procedure following the SAM study. Most participants recognized the potential of targeted strategies to support and enhance implementation. Suggested strategies included facilitating access to experienced tutors and providing a blueprint for implementation including material checklists, standardized surgery reports and patient information. An updated patient decision aid was not considered to offer substantial benefit by most participants.

## Discussion

In this study, we identified barriers and facilitators for the implementation of the MP in the Netherlands from gynecologists’ perspectives through semi-structured interviews. Our findings highlight strong regional collaboration, low complexity of the surgical technique, peer-driven motivation to align with national trends, and overall trust in the evidence as key facilitators, although the latter varied between participants. The most important barriers were the substantial personal and organizational effort required for training, particularly in the absence of experienced colleagues, as well as by satisfaction with SSH results, and concerns regarding lower reimbursement for the MP. These findings illustrate that adoption of the MP is influenced by a combination of clinical, organizational, and financial factors, and underline the need for implementation strategies that address both individual and system-level challenges.

A major facilitator for the MP adoption is the presence of regional collaboration networks among hospitals. These networks facilitate peer learning, informal mentorship, and practical knowledge exchange. Regular exposure to shared practices and expert discussions promotes alignment with evolving standards and encourages behavioral change. This facilitator reflects broader implementation science literature, which highlights the role of social influence and professional networks in practice change [17, 18]. While research on this topic in urogynecology is scarce, a Canadian study in gynecologic oncology shows that clinicians in regional structures, such as tumor boards or educational consortia, are more likely to adopt new practices when regularly exposed to expert discussions and peer norms [19]. This suggests that regional networks not only support the MP implementation in the Netherlands, but may also serve as a model for international adoption and continuous learning. In settings where centralized guidance is limited, fostering regional collaboration may be a practical strategy to advance evidence-based surgery.

Trust in the available evidence emerged as a central theme influencing clinical decision-making. While many gynecologists considered the current data sufficient to change practice, others remained hesitant, often citing perceived limitations in study design or lack of long-term outcomes. This shows that high-quality research can act as a powerful driver for change, yet some clinicians simply need more time or studies for change. Such hesitation may be shaped by past experiences, for example, the rapid and controversial introduction of mesh implants [20]. As Enklaar et al. [21] noted, practice variation in prolapse surgery has historically been driven by personal clinical experience in the absence of robust evidence. In contrast, the transition from uterus-removing to uterus-sparing procedures in Dutch clinical practice is hypothesized to have been driven by growing scientific evidence and is generally regarded as a successful shift, even though the implementation process was never formally studied [5]. While the evidence base in urogynecology has grown over time, long-term evidence remains scarce, and professional background continues to influence attitudes [3, 21, 22]. Implementation strategies should therefore combine evidence with clinical expertise, for example, through peer discussions.

To support such strategies, clinical guidelines may play a role in translating evidence into practice. Yet, guideline updates are often delayed, which can slow the adoption of new findings [23]. Moreover, guidelines alone don’t ensure implementation, as clinicians may wait for broader consensus or international endorsement before altering established routines [24, 25]. The SAM study received national attention and included patients from nearly half of Dutch centers, which supported its generalizability and facilitated adoption. Further uptake may be encouraged if organizations like International Urogynecological association (IUGA) endorse the MP more explicitly.

A financial barrier reported by some professionals was the current reimbursement structure in the Netherlands, in which SSH is reimbursed at a higher rate than the MP, despite comparable hospital costs. Our findings also revealed that not all gynecologists were aware of these reimbursement differences, which is consistent with previous reports of limited financial awareness among physicians in the UK and Ireland [26, 27]. Nevertheless, the imbalance might negatively influence physicians’ decision-making, or influence at the managerial level. This finding aligns with studies in robot-assisted surgery, where financial considerations were identified as significant barriers to adoption [28, 29]. Unfortunately, reimbursement structures are not easily changed, which underscores the importance of addressing economic factors early in the implementation process. To support implementation of the MP, addressing the current discrepancy in costs is crucial. Furthermore, highlighting the lower societal costs of the MP may be an effective strategy to encourage broader adoption, particularly in the context of rising healthcare expenses.

### Strengths and Limitations

The main strength of this study was the use of CFIR, allowing for a comprehensive and theory-informed exploration of barriers and facilitators. Second, the sample included a diverse group of gynecologists in terms of age, geographic region, and type of hospital, which enhanced the completeness and transferability of findings within the Dutch healthcare context. Third, the process of double interviewing and dual coding contributed to the credibility and reliability of the analysis. However, some limitations should be acknowledged. First, the sample size was relatively small (*n* = 12), which may limit the range of perspectives captured, even though data saturation was reached. Second, we initially faced challenges in recruiting gynecologists who did not perform MP or were unconvinced of its benefits. Ultimately, targeted efforts resulted in a balanced sample across different implementation stages. However, targeted recruitment and some nonresponses may have introduced selection bias, and it is possible that those with interest in the MP were more likely to participate. Additionally, responses related to financial incentives may have been influenced by social desirability bias. Finally, the study was conducted within the Netherlands, where clinical guidelines, healthcare organization, and financial structures differ from other countries. These contextual factors may influence both the perceived barriers and facilitators and the applicability of findings internationally.

To support successful implementation of the MP, it is essential to address the identified barriers and reinforce existing facilitators. Key strategies could include establishing mentoring structures to support training, developing practical materials to support clinical workflow, fostering peer discussions to build confidence in the evidence, and revising reimbursement models. These targeted efforts may promote broader adoption of the MP.

## Conclusion

In this study, we identified barriers and facilitators relevant for the implementation of the MP as first-line treatment in patients with mild to moderate uterine prolapse. Key facilitators included regional collaboration, the low procedural complexity, and peer-driven motivation to align with national practice trends. Barriers involved the demands associated with training and implementation, satisfaction with SSH outcomes, and financial concerns. Notably, trust in the available evidence functioned both as a facilitator and a barrier, depending on gynecologists’ interpretation of its robustness and relevance to their practice. Addressing these barriers and leveraging facilitators could enhance adoption of the MP, potentially improving treatment outcomes and reducing healthcare costs.

## Supplementary Information

Below is the link to the electronic supplementary material.

Supplementary file1 (DOCX 21 KB)

Supplementary file2 (DOCX 22 KB)

## Data Availability

The data that support the findings of this study are available from the corresponding author, L.M. Stoter, upon reasonable request.
