# Supplementary material for: Key Conditions for Successful Implementation of the Manchester Procedure as Primary Surgical Treatment for Mild to Moderate Uterine Prolapse: A Qualitative Study Among Dutch Gynecologists
Source: Int Urogynecol J. 2026 Feb 7;37(7):2153–61. doi: 10.1007/s00192-026-06525-7 (PMC13385254; doi:10.1007/s00192-026-06525-7)
Supplement: Supplementary file 1 — Supplementary file1 (DOCX 21 KB) [file 192_2026_6525_MOESM1_ESM.docx]

**Appendix I - Consolidated criteria for reporting qualitative studies (COREQ): 32-item checklist**

A checklist of items that should be included in reports of qualitative research. You must report the page number in your manuscript where you consider each of the items listed in this checklist. If you have not included this information, either revise your manuscript accordingly before submitting or note N/A.

| **No. Item** | **Guide questions/description** | **Reported on Page #** |
| --- | --- | --- |
| **Domain 1: Research team and reﬂexivity** |  |  |
| *Personal Characteristics* |  |  |
| 1. Interviewer/facilitator | Which author/s conducted the interview or focus group? | 4 |
| 2. Credentials | What were the researcher’s credentials? E.g. PhD, MD | LS: MD  EV: PhD  SD: PhD |
| 3. Occupation | What was their occupation at the time of the study? | LS was a PhD candidate at the Gynecology department, Radboudumc  EV was a post-doctoral researcher at IQ health, Radboudumc  SD was a senior researcher at IQ health, Radboudumc |
| 4. Gender | Was the researcher male or female? | All researchers were female |
| 5. Experience and training | What experience or training did the researcher have? | LS holds a Master’s degree in Medicine.  EV and SD both hold a PhD and have extensive experience in conducting qualitative research. |
| *Relationship with participants* |  |  |
| 6. Relationship established | Was a relationship established prior to study commencement? | No, there was no relationship established prior to the commencement of the study. |
| 7. Participant knowledge of the interviewer | What did the participants know about the researcher? e.g. personal goals, reasons for doing the research | Participants were informed that the researcher was also responsible for the broader implementation project under which this study was conducted. This was also described in the interview guide. |
| 8. Interviewer characteristics | What characteristics were reported about the interviewer/facilitator? e.g. Bias, assumptions, reasons and interests in the research topic | The interviewer was transparent about their role in the broader implementation project. No specific biases or assumptions were reported, but the interviewer had a professional interest in improving care related to the study topic. |
| **Domain 2: study design** |  |  |
| *Theoretical framework* |  |  |
| 9. Methodological orientation and Theory | What methodological orientation was stated to underpin the study? e.g. grounded theory, discourse analysis, ethnography, phenomenology, content analysis | 3 |
| *Participant selection* |  |  |
| 10. Sampling | How were participants selected? e.g. purposive, convenience, consecutive, snowball | 4 |
| 11. Method of approach | How were participants approached? e.g. face-to-face, telephone, mail, email | 4 |
| 12. Sample size | How many participants were in the study? | 4 |
| 13. Non-participation | How many people refused to participate or dropped out? Reasons? | One potential participant did not respond to the invitation of this study. There were no dropouts. |
| *Setting* |  |  |
| 14. Setting of data collection | Where was the data collected? e.g. home, clinic, workplace | 4 |
| 15. Presence of non-participants | Was anyone else present besides the participants and researchers? | 4 |
| 16. Description of sample | What are the important characteristics of the sample? e.g. demographic data, date | 4 |
| *Data collection* |  |  |
| 17. Interview guide | Were questions, prompts, guides provided by the authors? Was it pilot tested? | 4 |
| 18. Repeat interviews | Were repeat interviews carried out? If yes, how many? | No, there were no repeat interviews carried out. |
| 19. Audio/visual recording | Did the research use audio or visual recording to collect the data? | 4 |
| 20. Field notes | Were ﬁeld notes made during and/or after the interview or focus group? | No field notes were made during the interview. The interviews were digitally recorded and transcribed verbatim |
| 21. Duration | What was the duration of the inter views or focus group? | 5 |
| 22. Data saturation | Was data saturation discussed? | 4 |
| 23. Transcripts returned | Were transcripts returned to participants for comment and/or correction? | No, due to feasibility reasons, transcripts were not returned to the participants. |
| **Domain 3: analysis and ﬁndings** |  |  |
| *Data analysis* |  |  |
| 24. Number of data coders | How many data coders coded the data? | 5 |
| 25. Description of the coding tree | Did authors provide a description of the coding tree? | 5 |
| 26. Derivation of themes | Were themes identiﬁed in advance or derived from the data? | 5 |
| 27. Software | What software, if applicable, was used to manage the data? | 5 |
| 28. Participant checking | Did participants provide feedback on the ﬁndings? | No, they did not. However, the results of this study were presented at a large Dutch conference were there was room for discussion. |
| *Reporting* |  |  |
| 29. Quotations presented | Were participant quotations presented to illustrate the themes/ﬁndings? Was each quotation identiﬁed? e.g. participant number | 6-11 |
| 30. Data and ﬁndings consistent | Was there consistency between the data presented and the ﬁndings? | 11 |
| 31. Clarity of major themes | Were major themes clearly presented in the ﬁndings? | 11 |
| 32. Clarity of minor themes | Is there a description of diverse cases or discussion of minor themes? | 11-14 |
