# Supplementary material for: Key Conditions for Successful Implementation of the Manchester Procedure as Primary Surgical Treatment for Mild to Moderate Uterine Prolapse: A Qualitative Study Among Dutch Gynecologists
Source: Int Urogynecol J. 2026 Feb 7;37(7):2153–61. doi: 10.1007/s00192-026-06525-7 (PMC13385254; doi:10.1007/s00192-026-06525-7)
Supplement: Supplementary file 2 — Supplementary file2 (DOCX 22 KB) [file 192_2026_6525_MOESM2_ESM.docx]

**Appendix II - Interview Guide**

Implementation of the Manchester Procedure: Gynecologists' Perspective

Introduction

- Thank the participant for taking part in the study.
- Brief summary of the information letter:
  - Briefly explain the purpose of the study
  - Explain anonymity
  - Explain that the interview will be recorded to ensure no information is lost
  - Explain that the participant may stop the interview at any time
  - Ask if the participant has any questions
- Read the consent form aloud and ask for agreement

Background

As is well known, there is significant variation in the treatment of uterine prolapse in the Netherlands. The most commonly used uterus-preserving techniques at present are sacrospinous hysteropexy (SSH) and the Manchester procedure (MP). In 2023, the results of the SAM study were published, showing that the Manchester procedure yields better outcomes and is more cost-effective than sacrospinous fixation in women with mild to moderate uterine prolapse. Despite these findings, sacrospinous fixation remains the most frequently performed surgery in the Netherlands.

The aim of this interview study is to identify possible reasons for the limited adoption of the Manchester procedure. This will help us support hospitals in implementing the procedure.

Gynecologist Characteristics

- Gender: male / female
- Age: years
- Type of hospital: academic / teaching hospital / non-teaching hospital
- Training period: ………………………………………………………………………………………………
- Training region: ………………………………………………………………………………………………
- Surgical preference: Sacrospinous hysteropexy / Manchester procedure / no preference
- Number of prolapse surgeries in the past 12 months: …………………………………….
  SSH: ……………………………… MP: …………………………

Section 1: Characteristics of the Intervention

1. What is your personal view on the Manchester procedure (MP)? What are your experiences with MP? And with the alternative techniques SSH and vaginal hysterectomy (VH)?
   a. Where do these positive or negative experiences come from?
   b. What do you see as the advantages and disadvantages of MP compared to SSH?
   c. How important are these pros and cons to you when choosing a uterus-preserving surgery?
2. What is your opinion on the (quality of the) scientific evidence for MP? (e.g., the SAM study)
   a. Are you convinced of the added value of MP?
   b. Do you believe the results of the SAM study are sufficient to support a recommendation in the clinical guideline?
   c. To what extent are you still critical of the implementation of MP?
3. The current prolapse guideline dates from 2014 and is currently being updated. We expect that MP will be recommended for mild to moderate uterine prolapse. But what if the guideline update is delayed: would you already be willing to offer MP (more often) to patients?

Section 2: Involved Individuals – Gynecologists

1. Do you have a preference for MP or SSH? If so, why?
2. What is your perspective on learning and performing the MP procedure in clinical practice?
   i. Do you have sufficient knowledge about the procedure and its outcomes?
   ii. From whom did you learn the procedure?
   iii. Do you have sufficient (practical) expertise to perform MP?
   iv. Are you willing to perform both MP and SSH side by side?
   v. Do you train others in both techniques?
   vi. What are the most difficult steps to learn?
3. What do you consider to be the optimal ratio between SSH, MP, and VH in your clinic?
4. How important is a national implementation of MP in your opinion (with the goal of having at least 50% of first-time prolapse surgeries in each clinic be Manchester procedures)? Is this a necessity or a priority?
5. What do you personally expect to gain from it? (e.g., satisfaction, reward)
6. How do you make the decision for a uterus-preserving surgery during your consultation?
   a. What is your decision based on? (e.g., patient factors, patient preference, personal views, training, habits/behavior, colleagues’ opinions, departmental/group agreements, guidelines)
   b. Do you ever discuss both surgical options with your colleagues (within your department/from other hospitals)?
   c. What is the general opinion?
   d. If applicable, what do you teach others about relevant patient factors?

Section 3 – Inner Setting (Hospital)

1. Is the MP currently being offered by all urogynecologists in your hospital? What is the reason for this?
   a. To what extent does the size of the hospital play a role in this? (number of surgeries)
   b. To what extent does the hospital provide opportunities for training days? Is time and funding allocated for this?
2. To what extent has the hospital’s organization or structure played a role in the implementation?
   a. Are there agreements within the department or partnership that hinder or facilitate the implementation of MP? What is the general attitude of the partnership toward MP?
   b. To what extent is your hospital equipped to perform MP? (in terms of materials, facilities, staff, organization) *Note: provide explanation about required materials if needed.*
3. What would happen financially if you performed fewer SSHs and more MPs? Do you notice this personally? What does the partnership notice? What does the hospital notice? Does this play a role?
   a. What would change for you if the costs were equal? What would change if MP were better reimbursed than SSH? What is the optimal reimbursement ratio for you?
4. Is there a collaboration or network between you and urogynecologists in other hospitals?
   a. Would you be willing to act as a mentor in your region to help others learn MP? / Would you benefit from having a mentor in your region?
   b. Are there hospitals in your region that you believe need mentoring?
   c. Do you consider this important for the implementation of MP?
5. What innovations are taking place in pelvic floor care in your hospital?
   a. Do you see this as a barrier or facilitator for the implementation of MP?
   b. Sometimes it is difficult to change routines—do you think that might be the case here as well?

Section 4: Outer Setting (Dutch Healthcare System)

1. What do you believe is the role of the Dutch Society for Obstetrics & Gynecology in the implementation of MP?
   a. Is it important to you that new scientific findings are discussed at conferences, symposia, and meetings?
2. To what extent do you think social pressure from society plays a role in the implementation of MP? Is this a barrier or a facilitator?
3. Do you feel that all parties share the same goal, or are there conflicting interests that could potentially hinder implementation? (e.g., patients, gynecologists, hospitals, Dutch Society for Obstetrics & Gynecology, Dutch healthcare system, etc.)

Section 5: Involved Individuals – Patients

1. How do you counsel patients regarding uterus-preserving surgery during your consultation?
   a. How do you discuss the different uterus-preserving surgical options?
   b. Based on your experience, which factors are important to patients when making a decision?
   c. A patient preference study conducted before the publication of the SAM results showed equal preference for SSH and MP. The most important factors in decision-making were the likelihood of a successful outcome, the risk of dyspareunia, and the risk of buttock pain as a complication. Do you believe these are still the key factors for patients?
   d. How do you support the patient in making her decision? Do you provide a decision aid or refer her to one? Why or why not?
   e. To what extent does the gynecologist play a guiding role in the decision for or against MP?
2. Would anything change for you if you had to counsel patients for both MP and SSH? If so, what?
   a. What barriers do you see in this regard? (e.g., time, additional consultation, need for more reflection time)
   b. How could these be overcome? (e.g., patient preparation, decision aid)

Section 6: final question

- If you are not currently performing MP: Would you be willing to sign up for MP training tomorrow? Or are there things that need to be arranged beforehand or other factors that are holding you back?
- If you are already performing MP but could do more: Could you start offering MP to patients more frequently tomorrow? Or are there factors that are holding you back? What would prevent you from starting tomorrow?
